# Supplementary material for: The challenge of managing the commercial harvesting of the sea urchin Paracentrotus lividus: advanced approaches are required
Source: PeerJ. 2020 Oct 8;8:e10093. doi: 10.7717/peerj.10093 (PMC7548073; doi:10.7717/peerj.10093)

**Supplementary material**

**S1.** Results of General Linear Regression **(**GLM; AIC=473.72) with Gaussian distribution (Pearson normality test p-value = 0.2077) and model validation graphs for sea urchin density under commercial size in function of Sector and Habitat as fixed factors.

| **factor** | **Estimate** | **SE** | **t-value** | **p-value** |
| --- | --- | --- | --- | --- |
| intercept | 6.255 | 2.444 | 2.559 | 0.01264 |
| sector2 | -2.920 | 1.153 | -2.532 | 0.01354 |
| sector3 | -3.018 | 1.506 | -2.004 | 0.04889 |
| sector4 | -3.145 | 3.304 | -0.952 | 0.34448 |
| sector5 | -4.326 | 3.304 | -1.309 | 0.19466 |
| Calcareous rock | 6.278 | 2.362 | 2.658 | 0.00971 |
| Granite | 4.648 | 2.450 | 1.897 | 0.06186 |
| patchy meadow | -1.184 | 2.389 | -0.496 | 0.62158 |
| continuous meadow | NA | NA | NA | NA |


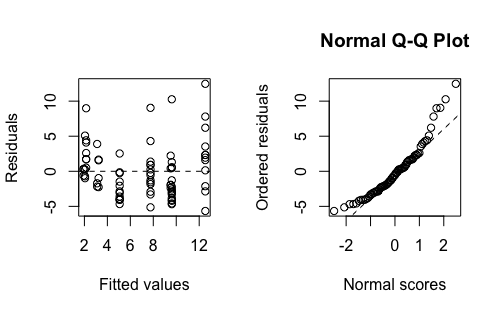


**S2.** Results of General Linear Regression **(**GLM; AIC=255.2) with Negative Binomial distribution and zero inflation (Pearson normality test p-value = 1.825e^-13^, percentage of zero 16%) and model validation graphs for recruit density in function of Sector and Habitat as fixed factors. Sector 5 was excluded from the analysis due to the perfectly multicollinearity between sector and habitat.

| **factor** | **Estimate** | **SE** | **z-value** | **p-value** |
| --- | --- | --- | --- | --- |
| intercept | -19.303 | 11258 | 0.0 | 0.999 |
| sector2 | -1.377 | 0.263 | -5.24 | 1.6e-07 |
| sector3 | -0.727 | 0.329 | -2.21 | 0.027 |
| sector4 | 20.284 | 11258 | 0.0 | 0.999 |
| Calcareous rock | 21.308 | 11258 | 0.0 | 0.998 |
| Granite | 0.491 | 0.452 | 1.09 | 0.277 |
| patchy meadow | 17.663 | 11258 | 0.0 | 0.999 |


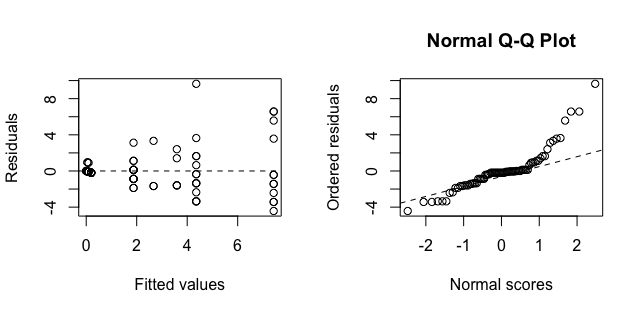


**S3.** Results of General Linear Regression **(**GLM; AIC= 376.64) with Gaussian distribution (Pearson normality test p-value = 0.1292) and model validation graphs for middle-sized sea urchin density in function of Sector and Habitat as fixed factors.

| **factor** | **Estimate** | **SE** | **t-value** | **p-value** |
| --- | --- | --- | --- | --- |
| intercept | 4.1253 | 1.5682 | 2.631 | 0.0104 |
| sector2 | 0.4806 | 0.7397 | 0.650 | 0.5180 |
| sector3 | -0.9503 | 0.9663 | -0.983 | 0.3288 |
| sector4 | -3.6919 | 2.1198 | -1.742 | 0.0859 |
| sector5 | -2.1919 | 2.1198 | -1.034 | 0.3046 |
| Calcareous rock | 2.2604 | 1.5154 | 1.492 | 0.1402 |
| Granite | 2.8667 | 1.5716 | 1.824 | 0.0724 |
| patchy meadow | -0.6193 | 1.5325 | -0.404 | 0.6873 |
| continuous meadow | NA | NA | NA | NA |

**
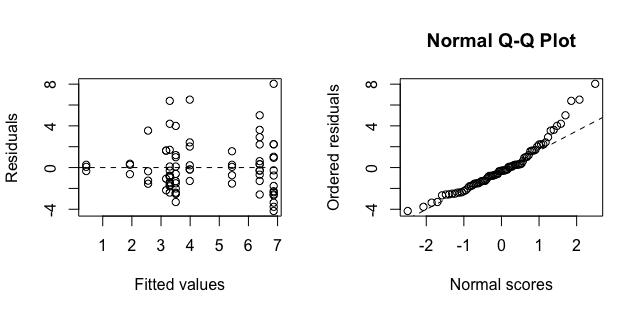
**

**Figure S4. GLM model validation graphs**


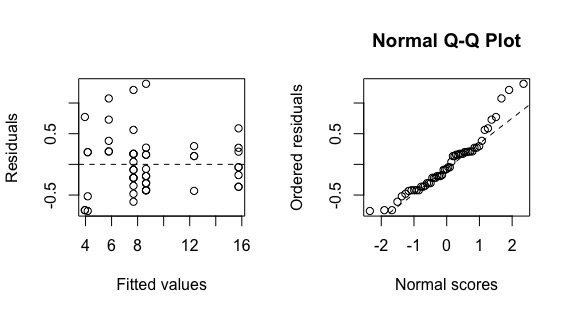

Supplement: Supplemental Information 1 [file peerj-08-10093-s001.docx]
